# Evolution of incidence and geographical distribution of Chagas disease in Mexico during a decade (2007–2016)

**DOI:** 10.1017/S0950268818002984

**Published:** 2018-11-13

**Authors:** G. Ibáñez-Cervantes, G. León-García, G. Castro-Escarpulli, J. Mancilla-Ramírez, G. Victoria-Acosta, M.A. Cureño-Díaz, O. Sosa-Hernández, J.M. Bello-López

**Affiliations:** 1 Escuela Superior de Medicina, Instituto Politécnico Nacional, Salvador Díaz Mirón, Col. Casco de Santo Tomas, 11340, México City, Mexico; 2 Hospital de la Mujer, SSA, Salvador Díaz Mirón 374, Col. Santo Tomas, 11340, Mexico City, Mexico; 3 Centro Médico y de Investigaciones Científicas Fundación CIAM ESPERAS, A.C., Felipe Carrillo Puerto 181, Col. Popotla, 11400, Mexico City, Mexico; 4 Departamento de Microbiología, Laboratorio de Investigación Clínica y Ambiental, Escuela Nacional de Ciencias Biológicas Instituto Politécnico Nacional, Carpio y Plan de Ayala, Col. Casco de Santo Tomás, 11340, México City, Mexico; 5 Hospital Juárez de México, Av. Instituto Politécnico Nacional 5160, Zacatenco, Gustavo A. Madero, 07360, México City, Mexico

**Keywords:** Chagas disease, epidemiology, incidence, México, *Trypanosoma cruzi*

## Abstract

Chagas disease, whose aetiological agent is the protozoan *Trypanosoma cruzi*, mainly occurs in Latin America. In order to know the epidemiology and the geographical distribution of this disease in Mexico, the present work analyses the national surveillance data (10 years) for Chagas disease issued by the General Directorate of Epidemiology (GDE). An ecological analysis of Chagas disease (2007–2016) was performed in the annual reports issued by the GDE in Mexico. The cases and incidence were classified by year, state, age group, gender and seasons. A national distribution map showing Chagas disease incidence was generated. An increase of new cases was identified throughout the country (rates from 0.37 to 0.81 per 100 000 inhabitants). Of the total cases accumulated (7388), the major cases were attributed to the states of Veracruz, Chiapas, Quintana Roo, Oaxaca, Morelos and Yucatán. The analysis per age groups and gender revealed that, in most age groups, the incidence was higher in the male population. The most number of cases was identified in spring and summer; a direct relationship between the environmental temperature increase and the number of new cases was identified. The analysis showed that the rate of Chagas disease increased presumably due to state programmes; the search for new cases has expanded and we speculate that the disease is associated with occupational activities. These results summarise and recall how important it is to implement the monitoring of Chagas disease mainly in south states of the Mexican Republic in order to implement strategies to control this disease.

## Introduction

Chagas disease, whose aetiological agent is the protozoan *Trypanosoma cruzi*, mainly occurs in Latin America; it is transmitted by bug bites of the genus *Triatoma*, *Rhodnius* and *Panstrongylus* [1]. The World Health Organization (WHO) estimated that, around the world ≈8 million people are infected and >25 million people, predominantly in Latin America, are at risk of the disease. Each year 100 000 new cases are reported, and 10 000 patients die annually in the world [2]. The disease is one of the most important emerging health problems in Europe and in the United States of America (USA) [3]. In this context, vector-borne diseases are one of the main causes of public-health problems in Mexico. Due to the geographic, climatic, demographic and socioeconomic characteristics of the Mexican Republic there is a potential risk of its transmission [4–6]. According to the National System of Epidemiological Surveillance, Chagas disease is not classified in the 20 causes of death in the country; however, it is a disease where the incidence is mainly located in the most vulnerable states, and it requires attention for its epidemiological surveillance. Epidemiological surveillance programmes analyse information of epidemiological importance such as infectious diseases, chronic diseases, risk events and emergency situations that put public health at risk, with the aim of implementing prevention and control actions. According to the National System of Epidemiological Surveillance, the identification of new cases or deaths associated with Chagas disease is subject to mandatory notification in accordance with the Official Mexican Standard for epidemiological surveillance (NOM-017-SSA2-2012) [7]. For the definitive notification of new cases, a laboratory algorithm must be performed in diagnostic tests. The primary laboratory diagnosis of the acute and chronic phase is performed through the demonstration of trypomastigotes in peripheral blood, molecular biology tests, blood culture or xenodiagnostic and infectious serology. All cases reported to the epidemiological surveillance system are subject to monitoring, which consists of monitoring or in some cases the possible conversion of indeterminate cases. Even when new cases of Chagas disease are notifiable, the National Epidemiological Surveillance System lacks information on the evolution of the incidence considering the geographic and demographic characteristics of the Mexican population. Due to all of the above, it is necessary to have evidence, with particular attention to the study of the epidemiological behaviour of this disease through the identification of risk factors and groups, the analysis of geographical distribution and the identification of possible outbreaks or new endemic areas. In order to know the epidemiological and geographical distribution of this disease in Mexico, the present work analyses the national surveillance data during the period 2007–2016 for Chagas disease issued by the General Directorate of Epidemiology (GDE). This information will also be helpful to promote prevention and to contribute to display the disease distribution in the last 10 years (from 2007 to 2016).

## Methods

### Period analysed and case definition

In this work, the incidence reports and new cases of Chagas disease from 2007 to 2016 were consulted and analysed. The corresponding data for 2017 is not yet available on the consulted website. The regulatory policies in Mexico established the mandatory notification of cases of Chagas disease. The Mexican territory is geographically distributed in a complex way; it is distributed in 31 states with a huge capital. This geographical complexity promotes the permanence of Chagas disease in states considered endemic. According to the Mexican Epidemiological Surveillance System, Chagas disease is classified as infectious, mainly transmissible vector, systemic parasitic disease and its classification is based on the standards established by the Pan American Health Organization, which defines a confirmed case when presenting double reactivity by third generation immunological techniques (chronic phase) or by demonstrating trypomastigotes in blood (acute phase). All confirmed cases must be notified weekly, the notification is regulated by the Official Mexican Standard (NOM-017-SSA2-2012).

### Data collection

The GDE receives the new reported cases of Chagas disease across the country on a monthly and annual basis. The collection and presentation of information was carried out under the observation of the principles of confidentiality and discretion outlined by the Federal Law of Accountability and Access to Public Government Information. The epidemiological data for the period 2007–2016 was taken from morbidity yearbooks found in www.epidemiologia.salud.gob.mx/anuario/html/anuarios.html. The data reported in this website was previously generated and analysed by the Health Department through its platform SUAVEweb (www.sinave.gob.mx). All data of new cases of Chagas disease (acute and chronic) was statistically analysed by using Microsoft Excel (Microsoft Corporation, Redmond, WA, USA) by month, year, states and demographic variables (age group and gender). Additionally, an incidence analysis of Chagas disease incidence by season was performed (per 100 000 habitants). An analysis of variance was also calculated in order to evaluate significant differences over time (*P* = 0.05), for data per year and month. A national distribution map showing Chagas disease incidence (per group) during the analysed period was generated. The incidence of Chagas disease during the 10 years analysed in the 32 states of the Mexican Republic was categorised into five groups (A, B, C, D and E), and was geographically mapped in the Mexican territory. Furthermore, an analysis of temporary trends of the incidence rates in the five groups previously mentioned was carried out. No ethical approval was obtained for the use of epidemiological information on Chagas disease in Mexico.

## Results

### Behaviour of new cases and incidence of Chagas disease

**Fig. 1.**
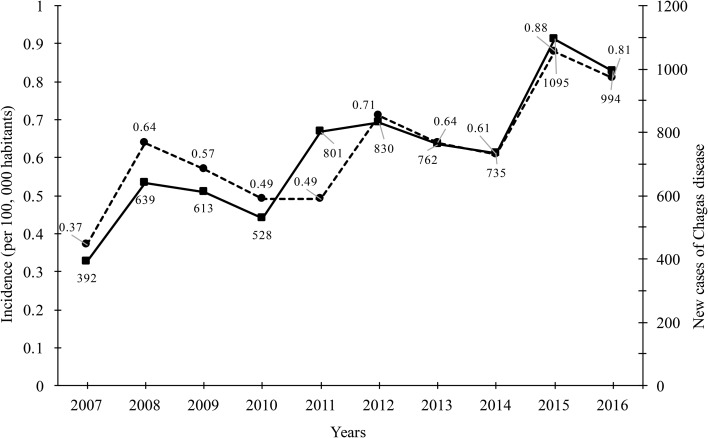
New cases of Chagas disease and incidence (per 100 000 habitants) in Mexican territory from 2007 to 2016.

During the period studied (2007–2016), a total of 7388 cases was reported, with a median of 738.8 cases per year (ranging from 392 in 2007 to 994 in 2016 per year), reflecting in an increase of 253.57% of new cases throughout the country (Fig. 1). Of the total cases accumulated during the period analysed, 58.52% (4324 cases) was attributed to the states of Veracruz, Chiapas, Quintana Roo, Oaxaca, Morelos and Yucatán. The remaining cases of Chagas disease (3064) were distributed in different proportions in the remaining 26 states of the Mexican Republic. A direct relationship between the number of new cases per year and the incidence (per 100 000 habitants) was observed. There is a significant increase in the incidence rates during the period analysed from 0.37 to 0.81. Throughout this period a median incidence of 0.62 ± 0.14 was identified.

### Geographical distribution of Chagas disease

**Fig. 2.**
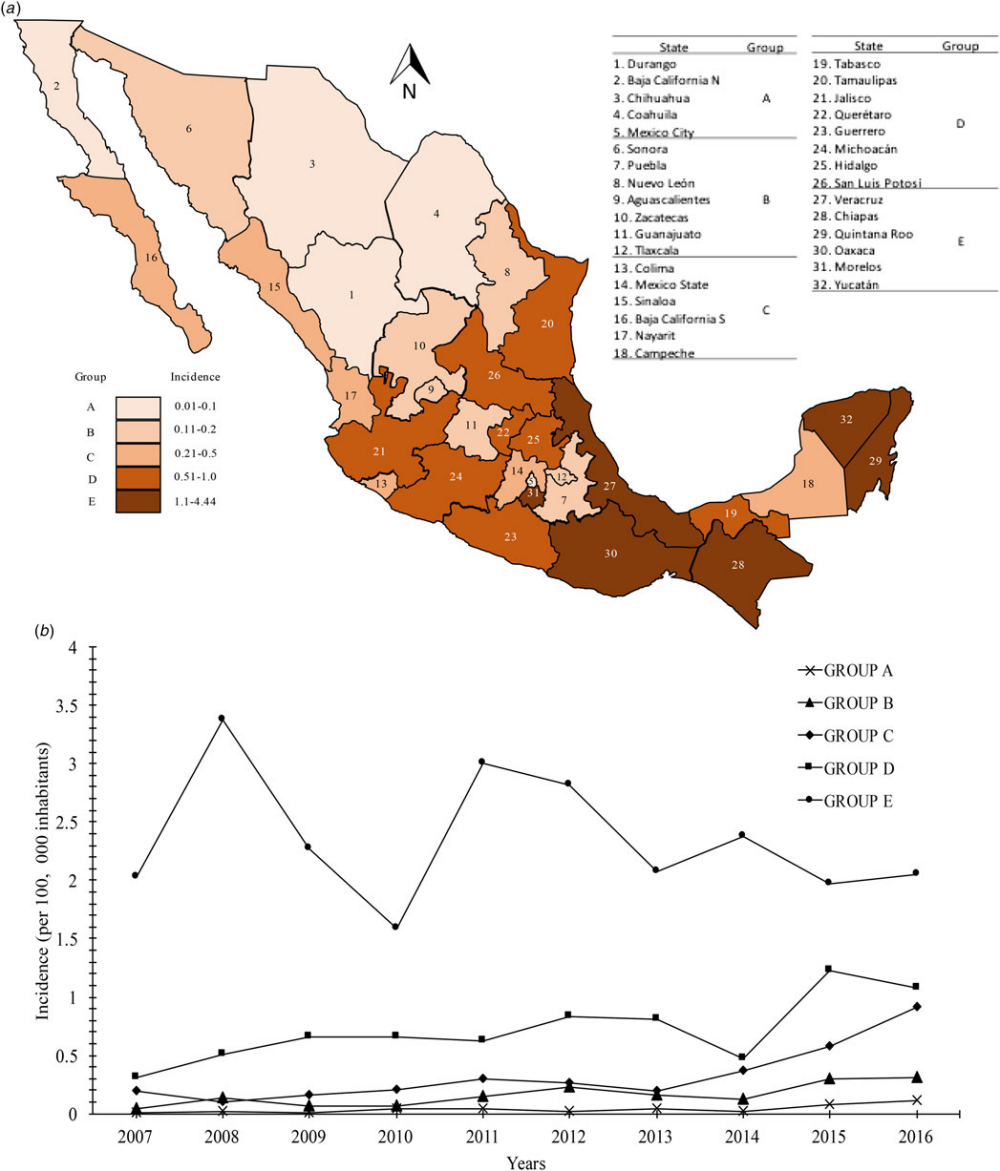
(a) Geographic distribution of the incidence rates (per 100 000 habitants) of Chagas disease by state in Mexican territory from 2007 to 2016. (b) Analysis of temporal trends of the incidence rates from 2007 to 2016.

The distribution of the incidence of Chagas disease all over the country is mostly headed by the states in the south Pacific coast (Gulf of Tehuantepec): Chiapas, Oaxaca (group E) with incidences of 1.1–4.4. Moreover, Veracruz, Quintana Roo and Yucatán, states belonging to the Gulf Coast of Mexico, were classified into group E. Interestingly, the state of Morelos, located in the central zone of the country was located in group E with an incidence of 2.59. Most of the states located in the central region of the country that presented incidences of 0.51–1.0 were located in group D (Tamaulipas, Jalisco, Querétaro, Guerrero, Michoacán, Hidalgo and San Luis Potosí). Although the state of Tabasco is located in the southeast region of the country, it was categorised in this same group with a mean incidence of 0.56. Finally, the remaining 18 states of the Mexican Republic were located in the last categories (A, B and C), which presented mean incidences of 0.01–0.1, 0.11–0.2 and 0.21–0.5, respectively. The states located in the northern region of the country (Durango, Baja California Norte and Coahuila) had the lowest incidences. Mexico City, located in the centre of the country, had an average incidence of 0.07 (group A) (Fig. 2a). Likewise, the analysis of temporal trends of the incidence rates in the five groups previously mentioned, confirms an increase in the incidence rates, with regards to the time in the five groups analysed (Fig. 2b).

### Incidence of Chagas disease per age group and gender

**Fig. 3.**
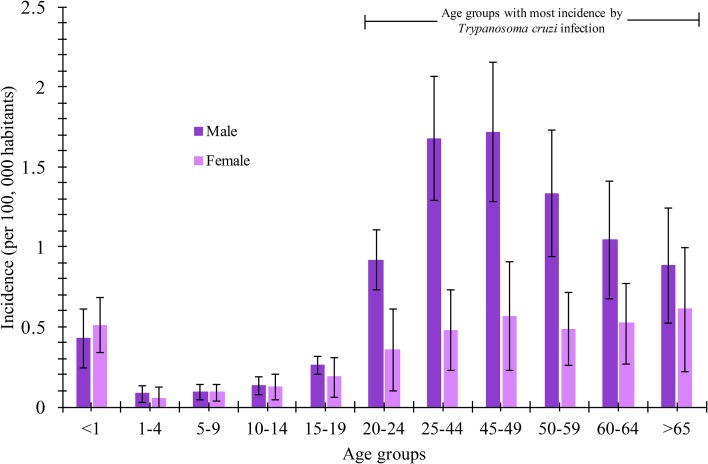
Incidence rate (per 100 000 habitants) of Chagas disease in Mexican territory by age groups and gender from 2007 to 2016.

The analysis of incidence (per 100 000 habitants) of Chagas disease by the age group showed that the male gender was the most frequent in the age groups of 20–24, 25–44, 45–49, 50–59, 60–64 and >65 years old with incidence values of 0.92 ± 0.18, 1.7 ± 0.38, 1.72 ± 0.43, 1.35 ± 0.39, 1.04 ± 0.36 and 0.9 ± 0.36, respectively. In contrast, the female gender presented incidences up to three times lower compared with the male gender in the age groups of 25–44 and 45–49 years old (0.567 *vs.* 1718 and 0.479 *vs.* 1678, respectively). The statistical analysis (*P* = 0.05) per age group and gender revealed that Chagas disease incidence was higher in the male population in most age groups (Fig. 3). Additionally, the maximum incidence peak was observed in the male gender in the interval between 25–44 and 45–49 years old. The mean national incidences were 0.77 and 0.36 for the male and female gender, respectively.

### Seasonal distribution of Chagas disease cases

**Fig. 4.**
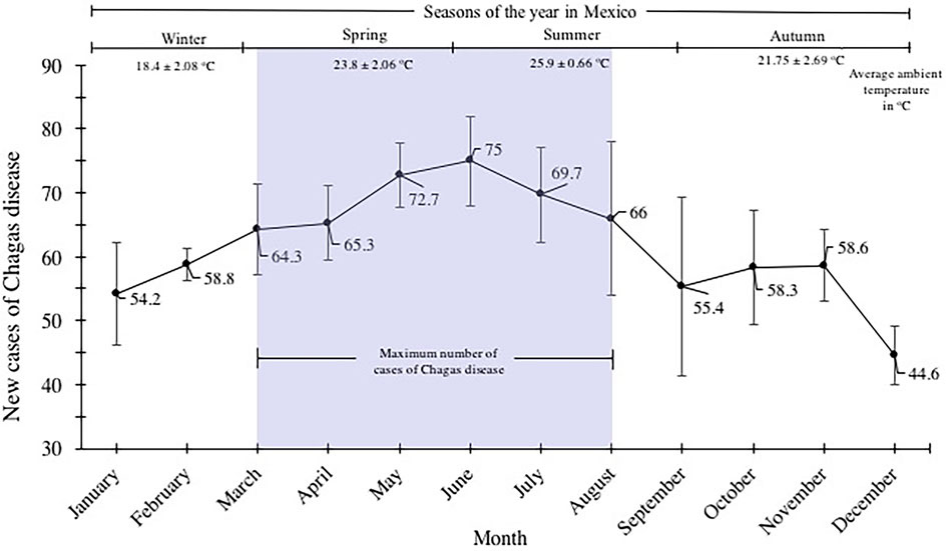
Seasonal variation of the mean of new cases of Chagas disease in the period 2007–2016 in Mexican territory.

New Chagas disease cases were categorised by month/year and were analysed by distribution within the yearly seasons (winter, spring, summer and autumn). The most number of Chagas disease cases was identified in spring and summer. The values of cases were 64.3 ± 7.2, 65.3 ± 5.8, 72.7 ± 5, 75.7 ± 7, 69.7 ± 7.5 and 66 ± 11 for March, April, May, June, July and August, respectively (spring–summer). A gradual decline of cases spanning the autumn and winter seasons was identified; values of 54.2 ± 8, 58.8 ± 2.6 of cases corresponded to the months of January and February; 55.4 ± 13, 58.3 ± 9, 58.6 ± 5.6 and 44.6 ± 4.6 for September, October, November and December, respectively (Fig. 4).

## Discussion

According to the National System of Epidemiological Surveillance, Chagas disease is not classified in the 20 causes of death in the country; however, it is a disease where the incidence is mainly located in the most vulnerable states of the country, and it requires attention for its epidemiological surveillance. In this work, it was demonstrated that Chagas disease continues to spread throughout the Mexican Republic, and it continues to be a national concern due to the increase in incidence in the last 10 years (Fig. 1). Annual increases in reported case rates may be due to the longitudinal improvements in surveillance and diagnostic capacities. As previously mentioned, the states located in the Pacific Coast (Gulf of Tehuantepec) and the Gulf Coast of Mexico were the most affected by the infection of this parasite (Fig. 2a). The analysis of the temporal trends of the incidence rates of the five groups in which the states of Mexico were divided shows a slight increase in the incidence rates with regards to the time (groups A, B, C and D), and incidences fluctuating with the incidence rates of group E (Fig. 2b). Previous studies have demonstrated that the southern states of the country have been a focal point due to the high incidence in the open population and in blood donors [8–11]. Furthermore, there are studies that indicate that there is no evidence of an excess risk of infection related to gender [12, 13]; however, in the present work, we identified that the most vulnerable groups of infection by *T. cruzi* were male subjects between 25–44 and 45–49 years old (Fig. 3). It is possible that the acquisition of the disease is strongly related to occupational activities since, in the same age groups in the female gender, the incidences were significantly different (*P* = 0.05). It is known that agriculture is one of the main occupational activities in the population of the endemic southern states; these activities could play an important role in the incidents identified, suggesting that the possible way of infection of *T. cruzi* in these regions was vector transmission in the working areas. Even though these data reflect the possible acquisition of the infection through occupational activities, it is necessary to have additional demographic information to prove this hypothesis. It is important to mention that the incidence in the population group under 1 year old (0.5) was significantly higher compared with the other age groups (up to 15–19 years old), this could be related to the increase of congenital transmission in recent years [14, 15]. Blood transfusion and blood components can be a transmission route of Chagas disease [16, 17], which is why, serological screening of blood donors has become very significant in the last decades. Mexico, as an endemic country, recognises serological screening of blood and blood components as compulsory for *T. cruzi* according to the Official Mexican Standard (NOM-253-SSA1-2012) [18], ‘For the provision of human blood and its components for therapeutic purposes’. Since a large number of seropositive cases has been identified in blood banks, studies about the incidence of Chagas disease in blood donors have demonstrated that the true extent of this infection is reflected in these blood collection sites, because the incidences of seropositive donations at the national level, as well as in some specific populations and in field studies, are significantly different from the cases reported outside of these transfusion centres [19, 20]. The latter clearly shows that blood banks could be opportunity areas to detect new cases, and thus, to estimate values close to the actual incidence in the general population. Regarding the analysis of geographical and seasonal distribution of the disease, an increase from March to August is observed, reaching a maximum peak in June, months of high temperature and heavy rains in the central and south parts of the country. A sharp decrease during the cold and dry months (September–December) was observed. These results reflect a direct relationship between the environmental temperature increase by season and the number of new cases of Chagas disease. Nowadays, little is known about the influence of temperature and altitude on the incidence of Chagas disease in our country. A previous study has reported that altitude could be an important factor in the development of the rectosigmoid length in Andean patients with Chagas disease [21], which suggests a direct relation between altitude and the virulence of the strain. Moreover, De Fuentes-Vicente *et al*. [22] measured in mouse models, the activity of phenoloxidase, key enzyme of defense response of triatomines, the level of parasitaemia and the number of amastigotes. In this study, it was reported that the evaluated parameters were significantly higher for infectious processes of *T. cruzi* from 700 m a.s.l., suggesting that this species could be more virulent than others. The influence of temperature, rain and altitude was reflected with greater incidences in the coastal states of Veracruz, Chiapas, Quintana Roo, Oaxaca and Yucatán, and in states of the central part of the country, such as Morelos which is located at 720 m a.s.l. in the southern part of the state; while, in the states with dry weather and short rainy seasons such as Durango, Chihuahua and Coahuila, incidences were lower. The National System of Epidemiological Surveillance suggests housing regulation and the use of residual insecticides, such as synthetic pyrethroids, to reduce risk and to control domestic infestation by vectors. This activity must be carried out before the rainy season, between February and May, since it is reported that, at this time of the year, the number of triatomine bites is higher, these data are consistent with the one reported in the present work. Torres-Montero *et al*. [23] have shown fluctuations in intradomiciliary infestation, with a maximum peak of presence of adult triatomines in June, month in which a greater number of cases was identified. Although, in this work, socioeconomic factors were not considered, in previous studies, these endemic areas have been identified with a high percentage of intradomiciliary and peridomiciliary infestation. This phenomenon is associated with precarious housing conditions with animal pens near the house [24–26]. Low incidences were reported in Mexico City and the urban area, this could be related to the habitat decrease of triatomines and better socioeconomic environments, compared with the endemic areas in the south part of the country. It has been suggested that the cases of Chagas disease in urban areas is related to migration from rural to urban areas [27, 28]. Results reported here can help to enrich the ‘Program of Specific Action to Prevent and Control Chagas Disease 2013–2018’ carried out by the Federal Government and the development of local and state situational diagnoses to assess risks of transmission and to implement a comprehensive prevention and control programme. Among the limitations shown in the present work, there could be: limited coverage of health services in the detection of Chagas disease, cases not reported by health institutions, cases not registered due to lack of assistance from the population for diagnosis and treatment, possible detection of false positive and negative cases in the diagnosis, among others. The analysis of the Chagas disease data during a decade shows that the situation of the disease in Mexico is understated, compared with the data reported in different research articles about seroprevalence of Chagas disease. It is important to mention that a large number of patients are chronic and without symptoms, without visible development of the disease and for which, clinical, epidemiological, laboratory and cabinet follow-up should be improved. The present analysis showed that the rate of Chagas disease increased, possibly because state programmes expanded their research for new cases; presumably the disease is associated with gender occupation activities. However, it is important to mention that the GDE does not consider the cases identified in blood transfusion centres; therefore, it is suspected that the incidences reported in the present work are higher. These results summarise and recall how important it is to implement the monitoring of Chagas disease cases in all instances of health where the detection is carried out in order to determine the current situation of this disease. Therefore, blood banks are opportunity areas to establish flowcharts of confirmatory studies and references to take care of confirmed cases.
